# Balance, Falls-Related Self-Efficacy, and Psychological Factors amongst Older Women with Chronic Low Back Pain: A Preliminary Case-Control Study

**DOI:** 10.1155/2012/430374

**Published:** 2012-08-09

**Authors:** Annick Champagne, François Prince, Vicky Bouffard, Danik Lafond

**Affiliations:** ^1^Department of Kinesiology, University of Montreal, Montreal, QC, Canada H3T 1J4; ^2^Department of Physical Activity Sciences, University of Quebec, Trois-Rivières, QC, Canada G9A 5H7

## Abstract

*Objective*. To investigate balance functions in older women and evaluate the association of the fear-avoidance beliefs model (FABM) factors with balance and mobility performance. *Participants*. Fifteen older women with CLBP was compared with age-matched pain-free controls (*n* = 15). *Main Outcome Measures*. Pain intensity, falls-related self-efficacy and intrinsic constructs in the FABM were evaluated. Postural steadiness (centre of pressure (COP)) and mobility functions were assessed. Linear relationships of FABM variables with COP and mobility score were estimated. *Results*. CLBP showed lower mobility score compared to controls. CLBP presented lower falls-related self-efficacy and it was associated with reduced mobility scores. FABM variables and falls-related self-efficacy were correlated with postural steadiness. Physical activity was reduced in CLBP, but no between-group difference was evident for knee extensor strength. No systematic linkages were observed between FABM variables with mobility score or postural steadiness. *Conclusions*. Back pain status affects balance and mobility functions in older women. Falls-related self-efficacy is lower in CLBP and is associated with reduced mobility. Disuse syndrome in CLBP elderly is partly supported by the results of this preliminary study.

## 1. Introduction

Back pain is among the most important factors affecting health status and functional capacity in elderly people [1], with a prevalence of 12 to 42% in subjects over 65 years of age [1]. It is more common in older women than in men [2], and women are more likely to have pain for prolonged periods [3]. Leveille et al. [1] determined that severe back pain increases the likelihood of disability by 3- to 4-fold, whereas mild or moderate back pain is not associated with reduced functional activities of daily living. Low back pain (LBP) was found to be related to 2-fold greater difficulty in daily living activities and the risk of falling [4]. Episodic chronic low back pain (CLBP) with advancing in age may cause neurophysiological changes that could further impact age-related deterioration of postural control [5]. 

According to the FABM [6], pain-related behaviours such as avoidance of physical activity and hypervigilance are consequences of catastrophizing misinterpretations or thoughts/beliefs triggered by pain experience. In young adults with CLBP, elevation of pain-related fear leads to heighten disability [7]. However, there is increasing evidence that pain-related fear does not systematically influence physical activity reduction or the emergence of the disuse syndrome [8–10]. In young adults with LBP, it seems plausible that pain-related fear provokes avoidance behaviours related to specific movements, that are believed to be potentially painful or at risk of reinjury, instead of impacting on general physical activity participation [8]. 

Nevertheless, constructs of the fear-avoidance beliefs model have not been studied extensively in older adults with LBP as in young adults. For example, Kovacs et al. [11] found that disability was not influenced by fear-avoidance beliefs related to physical activity in Spanish older adults. LBP elderly subjects reported more fear-avoidance and perceived less functional capacity, but did not show decreased physical activity compared to controls [12]. Moreover, fear-avoidance beliefs established using a specific-movement approach were found to predict functional capacity but was not a good predictor of physical activity participation [12]. High level of fear-avoidance beliefs was associated with falling [13, 14], whereas falling experience often contribute to decrease confidence in movement and in balance [15] in community-dwelling elderly individuals. Reduction of activities triggered by fear-avoidance beliefs also have an impact on physical abilities [13]. Despite the high prevalence of LBP in older adults and its influence on health-related outcomes, little information is available regarding the influence of LBP and fear-avoidance beliefs on balance and mobility functions as well as disuse syndrome in community-dwelling older persons. Important information could be gathered from direct measurement of balance and mobility functions in this population. In fact, pain-related fear has been associated with decreased physical function like reduced walking speed and lower-limb strength [16] in young adults with LBP. 

Therefore, the aims of this preliminary study are twofold: (1) to evaluate balance and mobility functions by direct measurements in a sample of older women compared with controls and (2) to examine if falls-related self-efficacy and constructs of fear-avoidance beliefs model are linked with poorer balance and mobility functions. We expect the following outcomes: (1) older adults with CLBP will have lower falls-related self-efficacy and (2) they will present lower balance and mobility functions. Although disuse syndrome has not been conclusively observed in elderly people with spinal pain [12], we maintain the following assumptions: (1) CLBP subjects will be less physically active and (2) falls-related self-efficacy, fear of movement, disability, and disuse outcomes will be linearly associated with balance and mobility functions in older adults with CLBP.

## 2. Materials and Methods

### 2.1. Participants

Fifteen CLBP women and 15 female controls without reporting pain, aged over 60 years, were recruited from the local community (Table 1). The controls were free of musculoskeletal pain during the last year and reported never having experienced disabling LBP. CLBP participants were included if they incurred LBP for at least 6 months, consecutively or episodically, and presented tension, soreness, and/or stiffness in the lower back region with radiating pain limited to the buttocks. The exclusion criteria were known causes of LBP, neurological or vestibular disease, dizziness, severe visual or hearing impairment, acute illness or pain, cognitive impairment, and medical conditions that could make testing potentially unsafe. Chronic health conditions and medications were screened from participant responses to interviewer-administered questionnaire, an extended version of the physical activity readiness questionnaire. Subjects answered “yes (1)” or “no (0)” when asked if they were presently suffering from health conditions or taking medications, and positive answers were summed. All subjects gave their informed, written consent according to the protocol approved by the University Ethics Committee (CER-10-156-04-02.01). 

### 2.2. Falls-Related Self-Efficacy

Participants were instructed to complete the Activities-specific Balance Confidence (ABC) Scale to assess falls-related self-efficacy [17]. They were also asked to recall their falls in the last 6 months. A fall was defined as any event that led to unplanned, unexpected contact with a supporting surface.

### 2.3. Back Pain Outcomes

On the day of testing, participants were told to rate back pain intensity on a quadruple numerical pain scale (QNPS) [18]. Functional disability related to LBP was evaluated with a modified French version of the Oswestry Disability Index (ODI) [19] calculated on 9 items without replacing the question about sexual activity by employment/homemaking ability [20]. Pain-related fear of movement was evaluated with the French version of the Tampa Scale for Kinesiophobia (TSK) [21].

The Baecke questionnaire (BPAQ) estimated physical activity level [22]. Only physical activity during sports and leisure time indexes were calculated; their summation provided an index of physical activity participation (PASL). The maximal isometric knee extensor (KE) force of each leg was estimated with a load cell in the seated position and the knee at 90°. Two 5-s trials of maximum isometric effort were conducted, and peak value was expressed as a relative strength index—a ratio of strength to body composition (body mass index, BMI) measurement. KE strength was averaged because no between-sides difference was found. PASL and KE strength were used to infer on disuse syndrome.

### 2.4. Sensorimotor Functions and Balance Functions

Several aspects of sensorimotor functions that affect balance, such as visual acuity, tactile sensitivity, and kinesthetic sense were studied [23]. Visual acuity was examined with a Snellen chart, and a Semmes-Weinstein-type pressure aesthesiometer assessed tactile sensitivity at the lateral malleolus of both ankles. Reposition error at the knees (extension) and trunk (flexion) were calculated during 5 trials. For each segment, subjects had to start in a neutral position, reach a target angle of approximately 15°, hold the position for 10 s, and return to the initial position. Absolute differences in degrees between the target angle and position reached (absolute error) were averaged. Variable error was considered to measure inconsistency. Segmental angles were calculated with an optoelectronic system (Optotrak Certus, NDI, Waterloo, Ontario, Canada).

Postural steadiness was evaluated by instructing the subjects to stand upright as still as possible, with eyes opened, on a force plate (OR6-2000, AMTI, Watertown, MA, USA) according to a procedure described elsewhere [24]. Center of pressure (COP) speed and frequencies were used to infer on postural steadiness. Cumulative power was calculated from consecutive frequency windows of 0.1-Hz width and expressed as percentages of total power between 0 and 4 Hz. It has been shown that visual, vestibular, and proprioceptive contributions on balance control are related to specific frequency bands of COP signals [25]. Total power at the 3 frequency bands was calculated: B1 = [0–0.1 Hz] for visual; B2 = [0.1–0.5 Hz] for vestibular; B3 = [0.5–1.0 Hz] for proprioception.

The Timed up and go (TUG) [26] and One-leg stance test (OLS) [27] were also administered. A practice trial was allowed, and 2 subsequent trials were averaged. Maximal walking speed was estimated by the incremental shuttle walk test [28]. Each of these tests (TUG, OLS, and walking speed) was attributed a score of 1 to 4 using quartiles of performance (1st quartile = 1 and 4th quartile = 4) from values proposed by Choquette et al. [29] and summed to generate mobility scores (0–12). TUG scores were reversed, with lower scores reflecting better performance.

### 2.5. Statistical Analysis

Data were explored for normal distribution and homogeneity of variances. Data that showed skewed distributions were log-10-transformed prior to inferential analysis and presented as median values with 25th–75th percentiles. Subject characteristics, psycho-behavioural variables, sensorimotor function measures, and disuse-related variables were compared among groups by independent *t*-test. We undertook one-way repeated-measures analysis of variance with directions as within-subject factors to compare groups for COP measures. Post hoc Tukey-HSD analyses were performed for significant *F*-tests. We considered confounding variables, such as age, BMI, medication, and number of chronic conditions. Age and sum of comorbidities were not retained as confounding variables because they were not correlated with any COP measures and mobility score. Analysis of covariance with LBP status as a between-subjects factor and BMI as covariate compared PASL. Univariate comparisons were made with Pearson correlation coefficients to determine the strength of associations between FABM-related variables with mobility score in the CLBP group. Partial correlations, correcting for medication, quantified linear relationships between pain level at its worst, and dependent variables. Statistical significance was set at *P* < 0.05, and all statistical analyses were conducted with STATISTICA software (Statsoft Inc., Tulsa, OK, USA).

## 3. Results

Subject characteristics are presented in Table 1. No significant between-group differences were observed for anthropometric characteristics and sensorimotor functions (visual acuity, tactile sensitivity, and repositioning errors at the trunk and knees).

CLBP subjects perceived lower falls-related self-efficacy than the controls (79.5 versus 93.5%) and showed more fear of movement/re-injury (43.8 versus 33.4). The level of perceived disability is relatively low (median = 15.4%).

Table 2 reports performance on 3 tests of mobility, the mobility score and disuse-related variables. Controls performed better on TUG and walked faster than CLBP subjects. No between-group difference was found for OLS time. The controls' mobility score was significantly higher than that of CLBP subjects. They were significantly more active than their CLBP counterparts but no significant between-group difference was apparent for KE strength.  Table 3 discloses the level of association between psychobehavioural variables in CLBP. ABC scores were linked with increased ODI scores. Falls-related self-efficacy showed slight nonsignificant correlations with pain level at its worst and PASL.

**Figure 1:**
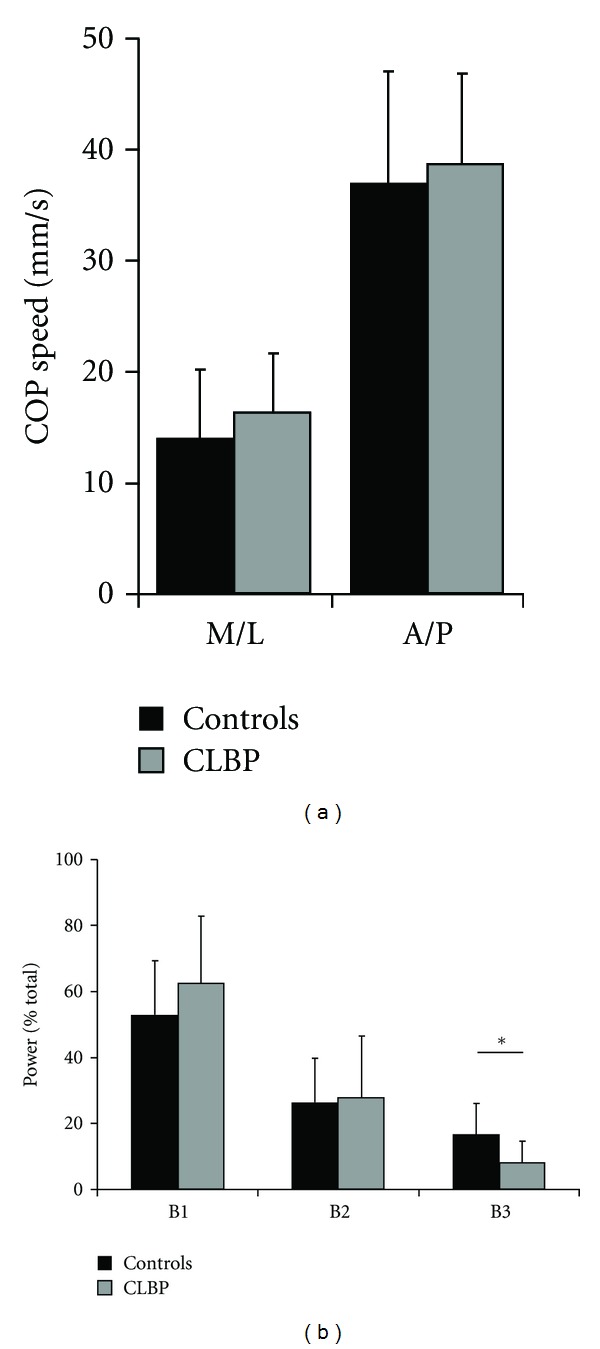
Comparison of postural steadiness between older CLBP women (grey) and controls (black). (a) Center of pressure (COP), speed in anteroposterior (A/P), and mediolateral (M/L) directions; (b) Power frequency bands (B1 to B3) for each group, expressed as a percentage of total power of COP signals. Data are mean ± SD; *P < 0.05.

No significant between-group difference was evident for COP speed, a measure of postural steadiness (Figure 1(a)).  Figure 1(b) illustrates the power of frequency bands (B1 to B3) for each group. CLBP manifested significantly decreased power at the B3 frequency band compared to the controls (*P* = 0.005). After correcting for BMI, B3 remained significantly different between groups in both anteroposterior (A/P) and M/L directions

Table 4 reports levels of association between psychobehavioural outcomes and disuse-related variables with COP speed and mobility score in the CLBP group. Mobility score was not correlated with any psychobehavioural outcomes and disuse-related variables (0.04 < *r* < 0.47). Pain level was significantly linked with COP speed. Falls-related self-efficacy was also related to COP speed in the M/L direction. No significant linkage was found between disuse-related variables and mobility score or COP speed.

## 4. Discussion

This preliminary study demonstrated that elderly CLBP displayed reductions in mobility performance and changes in postural control. It also highlighted associations of FABM outcomes and falls-related self-efficacy with balance functions after accounting for key covariates. Older women with low to mild back pain had lower falls-related self-efficacy linked with reduced mobility functions. Lower falls-related self-efficacy, decreased mobility functions, and diminished physical activity participation, observed in this study, could be important predictors of higher risk of falling and functional declines in CLBP women [1, 4].

The fear-avoidance belief model has been proposed to explain the development of CLBP [6]. Pain experiences are assumed to initiate fear of pain and fear of movement/reinjury that eventually lead to avoidance behaviours. Consequently, pain- and movement-related fear has been linked with decreased performance of physical tasks in young CLBP adults [16]. Reduction of physical capacities and activities as a result of avoidance behaviours is referred to as disuse syndrome [30]. However, disuse syndrome in young CLBP adults is a controversial issue [31]. Recently, Basler et al. [12] investigated the validity of the fear-avoidance belief model in a large sample of elderly CLBP and found no decline in PASL compared to the controls. They reported that fear-avoidance beliefs predicted physical disability but not PASL. In contrast, our results showed decreased PASL in elderly CLBP without a concomitant reduction of KE strength, as an indicator of physical deconditioning, compared to controls. Accordingly, Bousema et al. [32] noted a decline in physical activity without signs of physical deconditioning in young CLBP adults. We observed a significant correlation between perceived disability due to back pain and movement-related fear. However, in contrast to Basler et al. [12], PASL reduction was not associated with movement-related fear in the present study. This divergence in regards to decreased PASL in elderly CLBP could be explained by the fact that PASL was assessed with the Baecke questionnaire previously validated in CLBP subjects [22]. 

Fear-avoidance beliefs have been related to specific components of physical functions, such as knee extensors strength and COP displacements, in a sample of frail, elderly subjects, including fallers and multiple fallers [13]. Our sample of elderly CLBP did not experience frequent falls, which could explain why our data did not show a significant association between TSK with knee extensors strength and COP speed, respectively. Fear-avoidance beliefs in elderly CLBP may be correlated with greater disability, as seen in LBP young adults [33]. Our results did not disclose a significant association between fear-avoidance beliefs and back pain disability. Kovacs et al. [34] also discerned that fear-avoidance beliefs concerning physical activities were not related to back pain disability in elderly CLBP. In a larger sample of older Americans, Sions, and Hicks [35] established that fear-avoidance beliefs accounted for only 3% of variance of ODI scores, but high fear-avoidance beliefs were linked with greater falls experienced in the past year.

Self-efficacy, which is important in the maintenance of mobility in healthy ageing, is an independent factor of physical capacities [36]. The present study demonstrated a reduction of falls-related self-efficacy in elderly CLBP but no association was noted between falls-related self-efficacy and disuse-related variables. Balance impairments combined with weak muscular strength are assumed to be independent risk factors for falls or fractures [37], and knee extensors strength discriminates the ability to recover from gait perturbations to prevent falls [38]. In our study, knee extensors strength was not influenced by back pain status and was not correlated with COP speed or mobility score. Indeed, we did not see an association between falls-related self-efficacy and knee extensors strength. However, neither back extensor strength [39] nor endurance [40] were evaluated. Falls-related self-efficacy in elderly CLBP may be influenced by perceived capability concerning physical activities involving back movements. In support of this hypothesis, we observed a significant correlation (*r* = −0.60) between falls-related self-efficacy and perceived back disability. 

Some researchers have suggested that greater M/L sway increases the risk of falls [41]. We did not observe changes of postural steadiness in the M/L direction in elderly CLBP. Interestingly, falls-related self-efficacy was correlated with COP speed in the M/L direction. We noted a global decline of COP power spectral frequencies between 0.5 and 1.0 Hz that could be inferred to be a deficit in somatosensory input or integration for postural control [25]. Some authors have argued that pain may interfere in the somatosensory integration process or alter proprioceptive input acuity. This pain adaptation phenomenon is hypothesized to affect postural control [42]. Brumagne et al. [43] reported that LBP young people have reduced lumbosacral position sense that could be related to altered paraspinal muscle spindle afference or central integration problems. They also concluded that LBP elderly individuals may mostly rely on distal somatosensory input for postural control [44]. Consequently, we initially expected to find a systematic increase of postural sway and a significant decline of trunk and knee kinesthesia in elderly CLBP. In our study, however, back pain status does not influence joint repositioning senses and does not explain the COP frequency results. Goldberg et al. [45] witnessed an increase in trunk repositioning errors in elderly balance-impaired subjects. Those in the balance-impaired group were classified on the basis of One-leg stance time (<5 s). In our study, 4 CLBP and 2 controls had One-leg stance time <5 s. Trunk repositioning errors were assessed in upright stance by Goldberg et al. [45], whereas we have isolated trunk repositioning errors from postural control constraints. These differences in protocols may explain divergence of the results.

Higher fear of falling has been shown to be associated with activity restrictions and the increased likelihood of falling [46]. We saw a slight, nonsignificant correlation (*r* = 0.44) between falls-related self-efficacy and PASL. Sample size in this study and the retrospective design of falls assessment limited the detection of any linkage between falls occurrence and falls-related self-efficacy or activity restriction. Very few CLBP subjects had fallen during the previous 6 months of study entry. The use of retrospective self-reported falls might have led to underreporting [47]. However, declining physical activities cannot only be explained by a greater rate of falling because higher fear of falling and activity restriction have been encountered in older subjects who never experienced falls [48]. Another limitation is that pain duration was not assessed. However, having LBP with advancing age does not seem to be a cumulative phenomenon, and is more likely to be a disorder that originates from adulthood with persistence over 60 years [3]. Consequently, estimating pain duration may be difficult in elderly CLBP. Although the possible contribution of osteoporosis in back pain outcomes and falls-related self-efficacy cannot be excluded, vertebral fracture status could be a contributing factor in pain status rather than in balance and functional deficits [49]. We excluded clinically-relevant disease-related factors, such as diagnosed vertebral fracture, osteoporosis, and lumbar spinal stenosis. Depressive symptoms were not examined and could be a confounding factor for disabling LBP or falls-related self-efficacy.

In conclusion, our work, conducted among CLBP elderly women, highlights that falls-related self-efficacy is lower than in controls and is associated with reduced mobility functions. Postural control is affected by LBP status in older women and could be explained by alteration of somatosensory integration. These results may help clinicians to better manage CLBP in older patients and further highlight potential physical and psychobehavioural predictors of falls and functional decline in this population.

## Figures and Tables

**Table 1:** Subject characteristics.

	Controls	CLBP	*P* value
	*n* = 15	*n* = 15	
Age	69.4 (6.4)	68.9 (6.6)	.846
Body mass (kg)	67.5 (13.2)	72.2 (10.7)	.289
Height (m)	1.60 (0.06)	1.62 (0.08)	.447
BMI (kg/m^2^)	26.1 (4.4)	27.4 (3.7)	.390
ODI (%)	0 [0]	15.6 [13.3–24.4]	.000
TSK	33.4 (9.5)	43.8 (5.9)	.001
ABC	93.5 (4.6)	79.5 (17.2)	.005
Pain—low back region			
NPS-A	0 [0-0]	2 [1–4]	.000
NPS-W	0 [0-0]	7 [5–7]	.005
Chronic conditions	0.80 (0.77)	1.53 (1.19)	.055
Medications			
<4	11	11	.999
≥4	4	4	
Visual acuity	5.9 (1.0)	6.3 (0.9)	.262
Tactile sensitivity	4.1 (0.3)	4.0 (0.5)	.394
Repositioning sense (^°^)			
Trunk AE	1.4 [1.2–3.1]	1.5 [0.9–2.9]	.976
Trunk VE	0.6 [0.5–1.2]	0.4 [0.3–1.8]	.698
Knee AE	3.6 [2.5–5.6]	5.3 [2.9–8.1]	.403
Knee VE	2.6 [0.8–3.8]	2.4 [1.7–5.1]	.283

Data are mean (SD) or median [Inter-quartile range]. ODI: Oswestry disability index; TSK: fear of movement/reinjury; ABC: falls-related self-efficacy; NPS-A: averaged pain level; NPS-W: pain level at its worst; NPS-B: pain level at its best; BMI: body mass index; AE: absolute error; VE: variable error.

**Table 2:** Mobility functions and disuse-related variables.

	Controls	CLBP	*P* value
	(*n* = 15)	(*n* = 15)	
Timed up and go (s)	9.2 (2.0)	12.0 (3.6)	.012
One-leg stance (s)	12.0 (8.8)	10.9 (8.3)	.740
Walking speed (m/s)	1.66 (0.17)	1.43 (0.16)	.001
Mobility score (0–12)	8.9 (1.5)	6.9 (2.2)	.009
PASL	17.5 (4.9)	12.4 (5.3)	.012^∗^
F/BMI (N·kg^-1^/m^2^)	9.3 [7.6–11.2]	8.8 [7.3–10.8]	.611

PASL: physical activity participation; F/BMI: relative knee extensor strength index. ^∗^adjusted for BMI.

**Table 3:** Intercorrelations among psychobehavioural and disuse variables in the CLBP group.

	ODI	TSK	NPS-W	NPS-A	PASL	F/BMI
ABC	−0.60^∗^	−0.22	0.48^†^	−0.07	0.44	−0.12
ODI		0.34	−0.01^†^	0.34	−0.44	−0.21
TSK			−0.40^†^	0.35	0.12	0.35
NPS-W				0.26^†^	−0.18^†^	0.08^†^
NPS-A					−0.11	0.03
PASL						0.41

ODI: Oswestry disability index; TSK: fear of movement/reinjury; ABC: falls-related self-efficacy; NPS-W: pain level at its worst; NPS-A: averaged pain level; PASL: physical activity participation; *P < 0.05; ^†^partial correlation controlling for medication.

**Table 4:** Pearson correlation coefficients and partial correlations with mobility scores and COP measures (eyes open condition) for the CLBP group.

	Mobility score	COP speed	
		A/P	M/L
NPS-W			−0.77
NPS-A			−0.75^†^
ODI			
TSK			
ABC			−0.63
F/BMI			
PASL			

Only significant correlations are shown (*P* < 0.05). ODI: Oswestry disability index; TSK: fear of movement/reinjury; ABC: falls-related self-efficacy; NPS-W: pain level at its worst; NPS-A: averaged pain level; F/BMI: relative quadriceps strength index; PASL: physical activity participation; COP: center of pressure; A/P: anteroposterior direction; M/L: mediolateral direction; ^†^partial correlation coefficients adjusted for medication.
